# Incorporating Oral Health Considerations for Medication Management in Care Transitions

**DOI:** 10.3390/pharmacy8020067

**Published:** 2020-04-16

**Authors:** Kimberly A. Sanders, Christine L. Downey, Anita Yang, Brooke K. Baker

**Affiliations:** 1 Eshelman School of Pharmacy, University of North Carolina, 301 Pharmacy Lane, Chapel Hill, NC 27759, USA; anita23@email.unc.edu (A.Y.); brooke_baker@unc.edu (B.K.B.); 2 Adams School of Dentistry, University of North Carolina, 385 S Columbia St, Chapel Hill, NC 27599, USA; christine_downey@unc.edu

**Keywords:** transitions, oral health, medication management, readmissions, interprofessional practice

## Abstract

Transitions of care involve multifaceted considerations for patients, which can pose significant challenges if factors like oral health are overlooked when evaluating medication management. This article examines how oral health factors should be considered in medication management of patients who may be at risk for hospital readmission. This article also explores successes and challenges of a pharmacy consult service integrated into a dental clinic practice, and the opportunities within that setting to improve overall patient outcomes including those related to care transitions.

## 1. Introduction

Transitions of care can be challenging times for patients in ensuring they are well-informed and practicing health behaviors to prevent hospital readmissions. Access to follow-up care after hospitalization can be equally challenging to obtain and fraught with barriers. Approximately 17% of adults in the United States do not have a usual source of health care, including a primary care provider (PCP), and these statistics are less among racial and ethnic minorities [1]. While national efforts have focused on transforming health systems to increase collaboration and integration of services to address these shortages including efforts in care transitions, oral health has often been overlooked [2,3,4]. The dental setting has not traditionally been an access point for whole person care, yet twenty-seven million people visit a dentist annually in the United States who have not seen a PCP [5]. Chronic diseases impose a significant burden on the population and the public health infrastructure in terms of economic cost, disability, and death, and there is strong correlation between oral health and chronic illness [6,7,8]. This correlation, along with the lack of integrated services, perpetuate health disparities in care and access. 

## 2. Chronic Diseases and Oral Health

Chronic diseases such as cardiovascular disease, chronic obstructive pulmonary disease (COPD), and diabetes have been associated to both leading causes for hospital readmission and oral health-related complications [9,10,11,12]. While causality is not proven or clear, there is evidence of association between periodontal disease and chronic conditions. Periodontal disease is defined as a range of oral pathology from gingivitis that is curable and reversible to periodontitis that is irreversible [13]. Oral bacteria biofilms that lead to periodontal disease are reported to cause dysbiosis or imbalance in the microbiome of the mouth. As periodontal disease develops, it triggers the release of local pro-inflammatory cytokines that also play a significant role in systemic inflammation [13,14,15]. 

Specifically, in cardiovascular disease, the association between atherosclerosis and periodontal infection exists [10,13]. Systemic inflammation triggered by infection can increase cardiovascular stress and risk [16]. Oral bacteria may produce toxins with pro-atherogenic action as well. Lafon et al. noted that the risk of stroke was significantly increased by presence of periodontitis including a significant correlation with cardioembolic and thrombotic stroke types [17]. Support of preventative oral healthcare with the purpose of reducing biofilm to manage periodontal disease is important to reduce the risk of chronic inflammation that can then decrease the risk of stroke [18].

In regards to pulmonary diseases, like COPD, cytokines produced by periodontal inflammation can penetrate into the systemic circulation and can increase inflammatory burden in the lungs, worsening COPD [12,13]. Furthermore, the pathogens involved in chronic periodontitis are similar to pathogens involved in COPD. Manger et al. compiled evidence suggesting association between oral and pulmonary disease by relating periodontitis caused by bacterial infection to oral disease pathogens that can be aspirated into pulmonary tissues [12]. Without appropriate oral care, plaque formation can lead to colonization of virulent gram-negative pathogens that may transfer to the lungs to cause respiratory infection. 

What may be best defined and studied to date is the bidirectional relationship between diabetes and periodontal disease [19]. Hyperglycemia adversely impacts oral health through greater risk of alveolar bone loss, abscess formation, and poor wound healing, and severe periodontitis can negatively affect glycemic control. Periodontal infection and local inflammation can increase insulin resistance through the increase in systemic pro-inflammatory mediators, including cytokines [13,20,21]. Inflammatory cytokines influence intracellular pathways associated with insulin resistance in various cells throughout the body, including liver, muscle, immune system, and fat tissue; overall, making metabolic regulation more challenging in patients with diabetes and periodontal disease.

Therefore, in consideration of the associations described, great attention should be given to the medications used to treat not only these chronic conditions but review of medications that increase risk of dental caries and oral disease. Xerostomia is a common adverse effect of numerous medications that leads to impact on saliva production, changes in oral microbiome, and serves as a risk factor for caries and periodontal disease [22]. The microbiome of patients with periodontal disease also impacts the body’s regulation of metabolism. Given how the oral microbiome is significantly impacted by the level of oral hygiene, this is why awareness and prevention are crucial to reduce associated risks [23,24]. Additionally, noting that systemic condition treatment goals may be impacted by oral health and vice versa. 

It cannot be discounted that medication management for chronic conditions related to hospital readmissions can also be challenging for these populations due to access, affordability, lack of education, and lack of follow-up. Medication-related problems occur at a higher rate during care transitions [25]. These factors jeopardize and ultimately impact patients’ access and the optimal management of chronic conditions. In dental clinic settings, patients from rural areas with provider shortages may present with multiple chronic conditions; incomplete medical and medication histories; non-adherence or inappropriate medications; emotional distress; and inadequate food, transportation, or living arrangements. As a result, patients often experience suboptimal care of chronic diseases due to socioeconomic inequalities and the complexity of systems that limit the ability to provide equitable access to social and health resources. 

The University of North Carolina (UNC) Adams School of Dentistry (ASOD) has sought to better address these barriers within its practice clinics through clinical partnerships with the UNC Eshelman School of Pharmacy (ESOP) to offer clinical pharmacy services as it has identified patients in North Carolina who come from 74 out of the 100 counties that are dental provider shortage areas, and 59 of these counties are primary care and behavioral health shortage areas as well [2]. Pharmacists contribute significantly to patient outcomes in health care environments and have also begun to explore models in dental practice [26,27,28,29,30,31].

## 3. Clinical Service Creation

Placement of a clinical pharmacist in the dental practice clinics at UNC ASOD originated out of a dental student research project that focused on diabetes care of patients coming to the dental school for treatment. The intent of the project was to provide more wholistic education to the patients about diabetes given the link to periodontal disease as described. In usual care, the dental students provided cleanings, procedures, and counseling on appropriate oral hygiene in between appointments such as use of proper brushing and flossing technique, interdental cleaning aids, and oral irrigators [24,32,33]. The project added services to the appointment including a nutritionist who provided dietary counseling, and a pharmacist who provided medication education and monitoring to the patients in conjunction with the dental appointment. Upon observation of the patient population seen, the pharmacist noted other significant chronic conditions that would implicate multiple medications in reviewing and consulting on other patients, but the medication lists in the health record were frequently inaccurate due to issues such as omissions, incomplete dosing, and duplications. One example of a health record noted a patient who recently had a stroke and was hospitalized, but the medication list was not updated to include the new medications expected from a hospital discharge for such a condition including changes to the patient’s anticoagulant regimen.

Support from ESOP and ASOD in 2016 led to creation of a clinical pharmacist faculty position to provide more in-depth consultation services to help bring awareness to gaps stated above. As services expanded, consult services were conducted by three pharmacists and additional Doctor of Pharmacy students that included reviewing patient medication histories and profiles, providing medication and disease state education, documenting on medications impacting oral health, counseling on prevention and treatment of medication-related oral manifestations, and answering medication-related inquiries.

A pharmacy consultation guidance document was also created to help dental providers and students recognize when a pharmacist should be consulted and included six types. In describing the six types, it is important to note that consultations primarily occurred in conjunction with dental appointments. Patients received their dental care and education on preventative oral hygiene practice from the dental team as described above, while pharmacy consultations were integrated based on consult type. 

Consult type one involved a full medication history verification and reconciliation by a pharmacist and pharmacy student. Generally, this was for patients on complex regimens who were coming for initial diagnosis and treatment planning dental appointments. Consults for patients who could not recall what medications they were taking were also requested through this category. The patient’s dispensing pharmacy was identified and contacted to verify their regimen and clarify missing or incorrect doses. Additionally, medication regimens were evaluated for oral health implications, including but not limited to medications causing xerostomia that increases caries risk and medications that increase bleeding risk for invasive procedures. Patient and provider education was provided at point of consult and documented in the electronic health record. 

Consult type two involved in-depth assessment of high risk medications including anticoagulants and insulin. Indications and last doses taken of anticoagulants were confirmed to ensure safety of procedures prior to oral surgery.

Consult type three consisted of evaluation of patients with chronic conditions of hypertension, diabetes, asthma, COPD, and/or smoking to determine overall disease control and safety for procedures. Opportunities to educate on lifestyle management to help in prevention and maintenance were also provided.

Consult type four consisted of evaluation of patients with other significant conditions or medications that directly impact oral health including bisphosphonate use, gastroesophageal reflux disease (GERD), antimicrobials, antipsychotics, antineoplastic medications, and history of radiation. Education on administration and adverse side effects were primarily provided to help promote understanding and reduce risk of oral health complications. 

Consult type five involved providing patient education for any new medications prescribed within the scope of the dental care. This primarily included discussion of antibiotics, antifungals, analgesics, anesthetics, saliva modifiers, and anxiolytics with dental providers and students. Thorough review of a patient’s existing medication list was conducted to identify potential drug-drug interactions and adverse effects. Medication education was offered to patients prior to end of appointment to provide overview of dosing instructions and adverse side effects. 

Consult type six involved responding to any specific drug information questions related to medications requested by dental providers or students or voiced by a patient directly. Verbal or written responses were provided and documented in the electronic health record.

## 4. Successes

The clinical pharmacy consultation service continues to expand and evolve, and there have been many successful endeavors and teaching opportunities to overall improve patient care and understanding of links between oral health and systemic health. The initial pilot of pharmacy consult services included collecting dental student perspectives on roles of pharmacy personnel in dental care and interprofessional communication. Focus groups with students also highlighted an increase in integration of clinical discussion of medications and complex cases within the didactic curriculum.

Involvement of pharmacy students has also expanded. Additional toolkits were developed to help with flow of the consult process within the clinic space for pharmacy students rotating through the clinical environment as part of their experiential curriculum. A sit-down rounds model with pharmacy and dental students was also implemented to provide clinical teaching and deeper discussion of patients with complex medication regimens. Increased focus on opioid prescribing in dental pain management and antimicrobial stewardship have also garnered more significant attention within the clinical settings to address optimizing current and future prescribing behaviors.

Incorporation of a clinical pharmacist faculty position within the dental school faculty has also served as a framework for other health disciplines to integrate into the dental practice clinics including a social worker and nurse practitioner to provide enhanced team-based patient care.

## 5. Challenges

While there has been significant success in integration of a pharmacy team within the dental practice clinics, challenges still exist to further optimize patient care. Electronic health records (EHRs) are not linked to many primary care or health care centers. Progress is being made in implementing a broadly accepted EHR system within the dental school that is linked to the larger health system nearby. This still poses challenges for patients who travel from rural communities or far distances and do not have PCPs or routine medical care. Many patients without dental insurance coverage are also older adults with more complex health histories. The dental practice clinics serve as an entry point to the health care system, and strides are being made to better connect those patient populations with health care access back in their local communities. 

## 6. Future Direction

While oral health has been historically siloed from overall medical care, due to the links between chronic conditions and oral diseases, increased focus and attention should be made to incorporate oral health assessment, especially in patients recently discharged from hospitalizations or transitioning between other care environments like long-term and skilled nursing care facilities. Patients with poor oral health may be in jeopardy of increased risk of hospital readmission. Better coordination of care between these environments is crucial. Future expansion of services to include not only pharmacy but social work and advanced practice nursing will aid in this endeavor.
